# *catena*-Poly[[(8-amino­quinoline)­cobalt(II)]-di-μ-azido]

**DOI:** 10.1107/S2414314624008496

**Published:** 2024-09-06

**Authors:** Fatima Setifi, Zouaoui Setifi, David K. Geiger, Mohammed Hadi Al-Douh, Abderezak Addala

**Affiliations:** a Laboratoire de Chimie Ingénierie Moléculaire et Nanostructures (LCIMN) Université Ferhat Abbas Sétif 1 Sétif 19000 Algeria; b Département de Technologie Faculté de Technologie Université 20 Août 1955-Skikda BP 26 Route d'El-Hadaiek Skikda 21000 Algeria; c Department of Chemistry, SUNY-College at Geneseo, Geneseo, NY 14454, USA; d Chemistry Department, Faculty of Science, Hadhramout University, Mukalla, Hadhramout, Yemen; Goethe-Universität Frankfurt, Germany

**Keywords:** crystal structure, cobalt(II), 8-amino­quinoline (8-aquin), azide anion

## Abstract

The title coordination polymer, [Co(N_3_)_2_(C_9_H_8_N_2_)]_*n*_, was synthesized solvothermally. The Co^II^ atom exhibits a distorted octa­hedral [CoN_6_] coordination geometry with a bidentate 8-amino­quinoline ligand and four azide ligands. Bridging azide ligands result in chains extending along [100]. N—H⋯N hydrogen bonds join the chains to give an extended structure with sheets parallel to (002).

## Abstract 2

[Co(C_9_H_8_N_2_)(N_3_)_2_]_*n*_ exhibits a distorted octa­hedral coordination geometry. Bridging azide ligands result in chains along [100].

## Graphical abstract

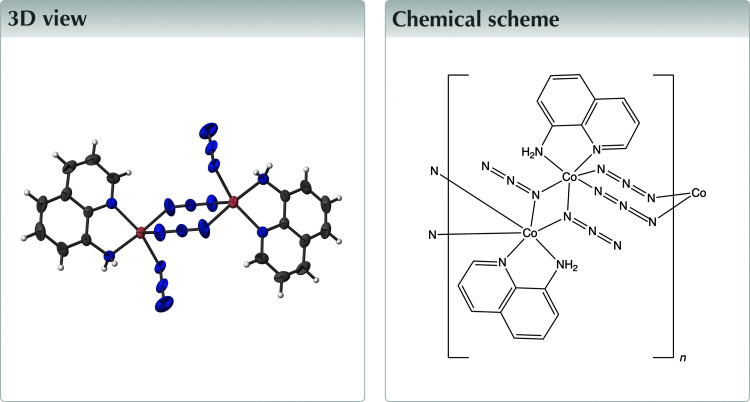

## Structure description

Pseudohalide and polynitrile compounds derived from transition-metal ions are of great inter­est from the perspective of their magnetic properties, rich mol­ecular architectures and for their topologies (Atmani *et al.*, 2008 ▸; Benmansour *et al.*, 2008 ▸, 2010 ▸, 2012 ▸; Addala *et al.*, 2015 ▸; Setifi *et al.*, 2018 ▸, 2019 ▸; Dmitrienko *et al.*, 2020 ▸; Yuste *et al.*, 2009 ▸; Merabet *et al.*, 2022 ▸).

One of the pseudohalide ligands that has received much attention in the last decade is the azide [N_3_^−^] ion, partly due to its ability to produce a wide variety of coordination compounds with different nuclearities ranging from simple mononuclear to polynuclear species. Different bonding modes are observed with the azide ion, which result in the formation of one-, two- and three-dimensional polymeric assemblies (Escuer *et al.*, 2006 ▸).

As a part of our continuing study of the structural and magnetic properties of transition-metal complexes containing both azide and polypyridyl units (Setifi, Ghazzali *et al.*, 2016 ▸; Setifi, Knaust *et al.*, 2016 ▸; Setifi, Moon *et al.*, 2016 ▸; Benamara *et al.*, 2021 ▸; Merabet *et al.*, 2023 ▸; Setifi, Setifi *et al.*, 2022 ▸, 2023 ▸), we report herein the crystal and mol­ecular structure of a one-dimensional coordination polymer, (I), based on 8-amino­quinoline (8-aquin) as co-ligand and the azide anion as ligand with two different coordination modes.

**Figure 1:**
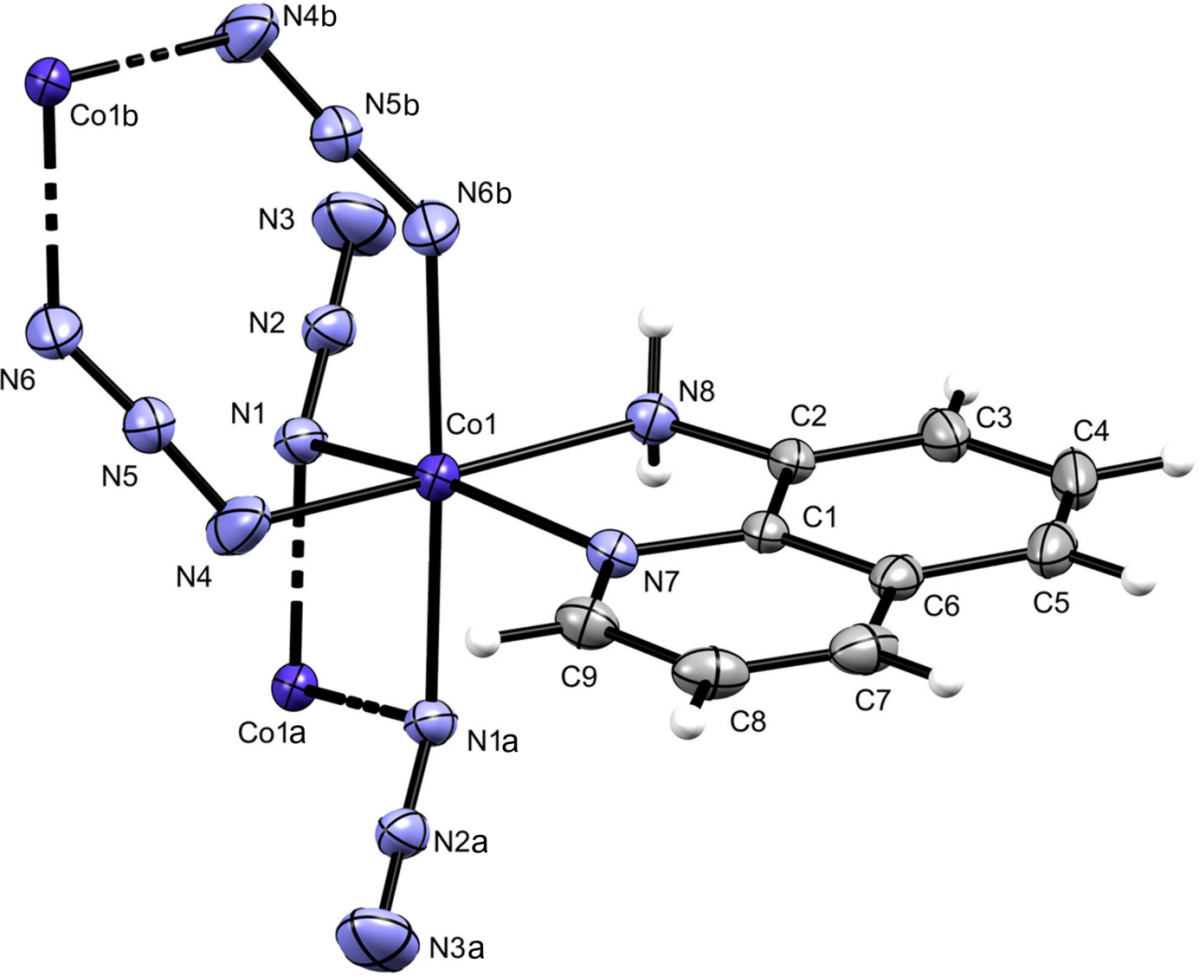
Representation of (the title compound showing the atom-labeling scheme. Non-H atom anisotropic displacement parameters are represented at the 50% probability level. Symmetry codes: (*a*) −*x*, −*y* + 1, −*z* + 1; (*b*) −*x* + 1, −*y* + 1, −*z* + 1.

The asymmetric unit of (I) is composed of a Co^II^ ion, a bidentate 8-aquin ligand and two azide ligands. The distorted octa­hedral coordination sphere is completed by two additional azide ligands. One of the azide anions binds to two Co centers in a 1,3 bidentate mode, whereas the other one connects two Co centers in a 1,1 bidentate mode. The resulting coordination geometry and supramolecular association is shown in Fig. 1 ▸. Pertinent Co—N bond lengths are exhibited in Table 1 ▸.

**Figure 2:**
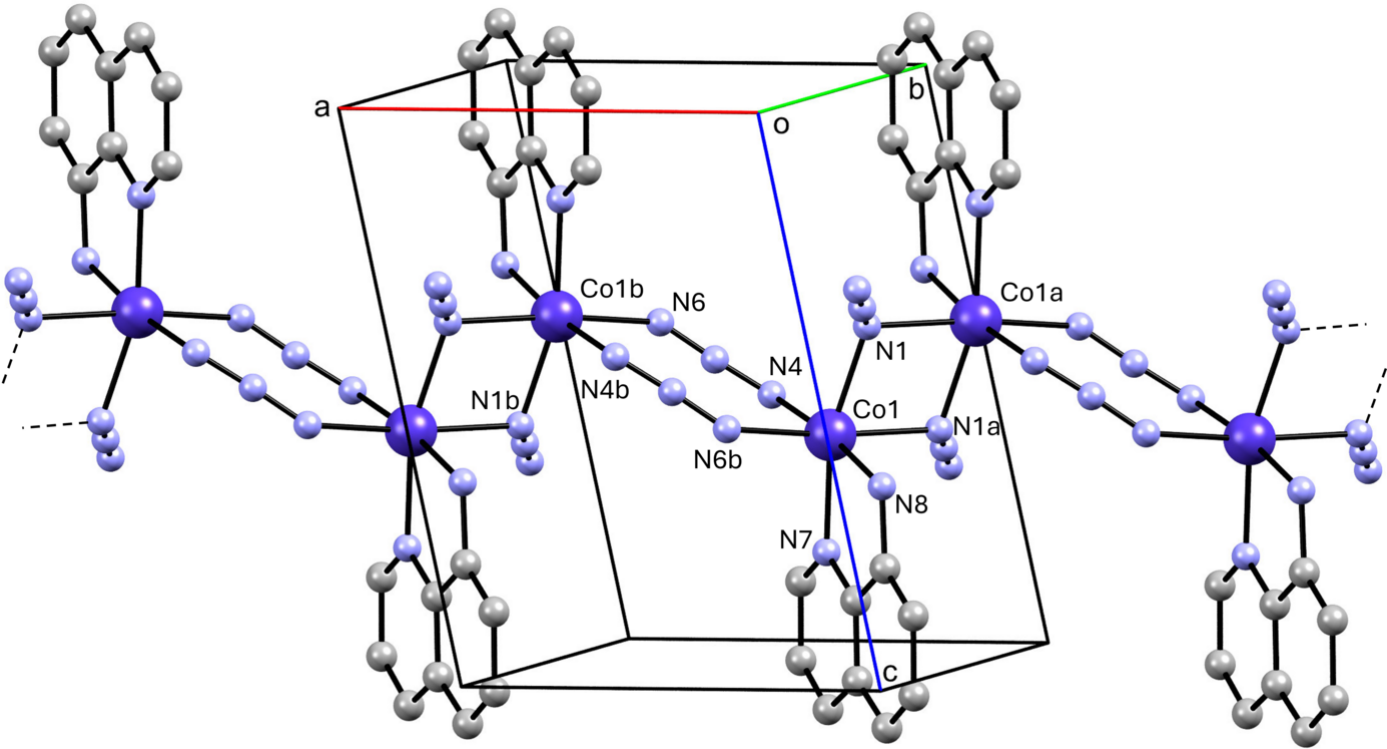
Partial packing diagram showing the polymeric chain parallel to [100]. H atoms are not shown. Symmetry codes: (*a*) −*x*, −*y* + 1, −*z* + 1; (*b*) −*x* + 1, −*y* + 1, −*z* + 1.

The bridging ligands result in polymeric chains extending parallel to [100], as shown in Fig. 2 ▸. The chains are composed of Co^II^ ions joined by alternating bis μ-(1,1-N_3_) units and bis μ-(1,3-N_3_) units with corresponding Co⋯Co separations of 3.2817 (5) and 5.2427 (7) Å, respectively. The angle between the (N_3_)_2_ mean plane of the double *end-to-end* azide bridges and the plane defined by the Co^II^ and the bonded N_azide_ atom is 20.20 (14)°, corresponding to a flattened chair configuration for the eight-membered ring. For a flat bridge, an angle of 0° would be observed. This angle compares to values of 8.2 (2) and 25.6 (1)° for the structurally similarly bridged polymorphic Fe^II^ complexes with a 5,5′-dimethyl-2,2′-bi­pyridine ligand (Setifi, Bernès *et al.*, 2022 ▸). In the 8-acquin complexes of Mn^II^ and Co^II^, the comparable angles are 20.3 (6)° and 25.5 (4)°, respectively (Benamara *et al.*, 2021 ▸).

**Figure 3:**
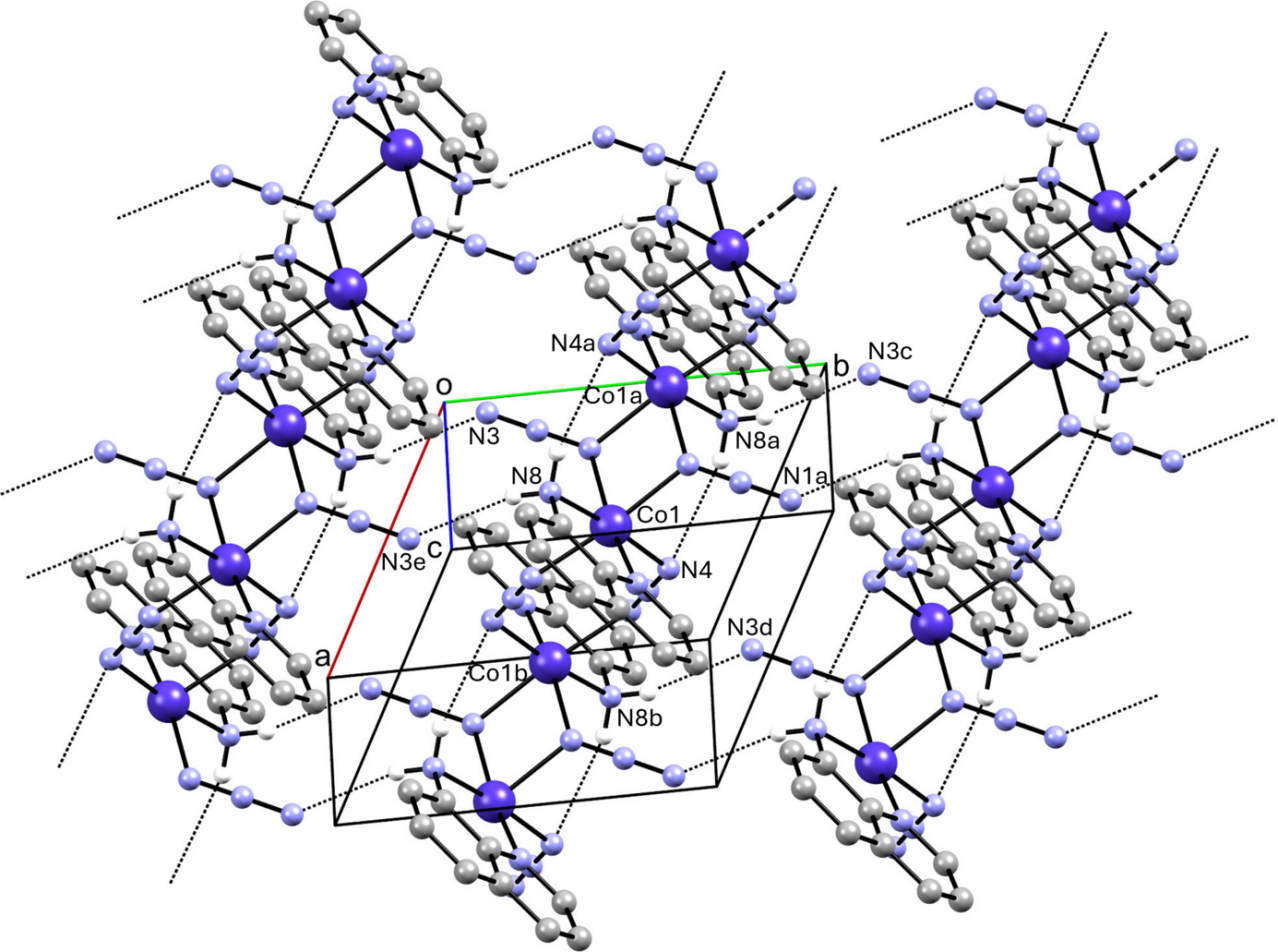
Partial packing diagram showing sheets parallel to (002) formed by N—H⋯N bonds. Only H atoms involved in the inter­actions are represented. Symmetry codes: (*a*) −*x*, −*y* + 1, −*z* + 1; (*b*) −*x* + 1, −*y* + 1, −*z* + 1; (*c*) *x*, *y* + 1, *z*; (*d*) *x* + 1, *y* + 1, *z*; (*e*). −*x*, −*y*, −*z* + 1.

**Figure 4:**
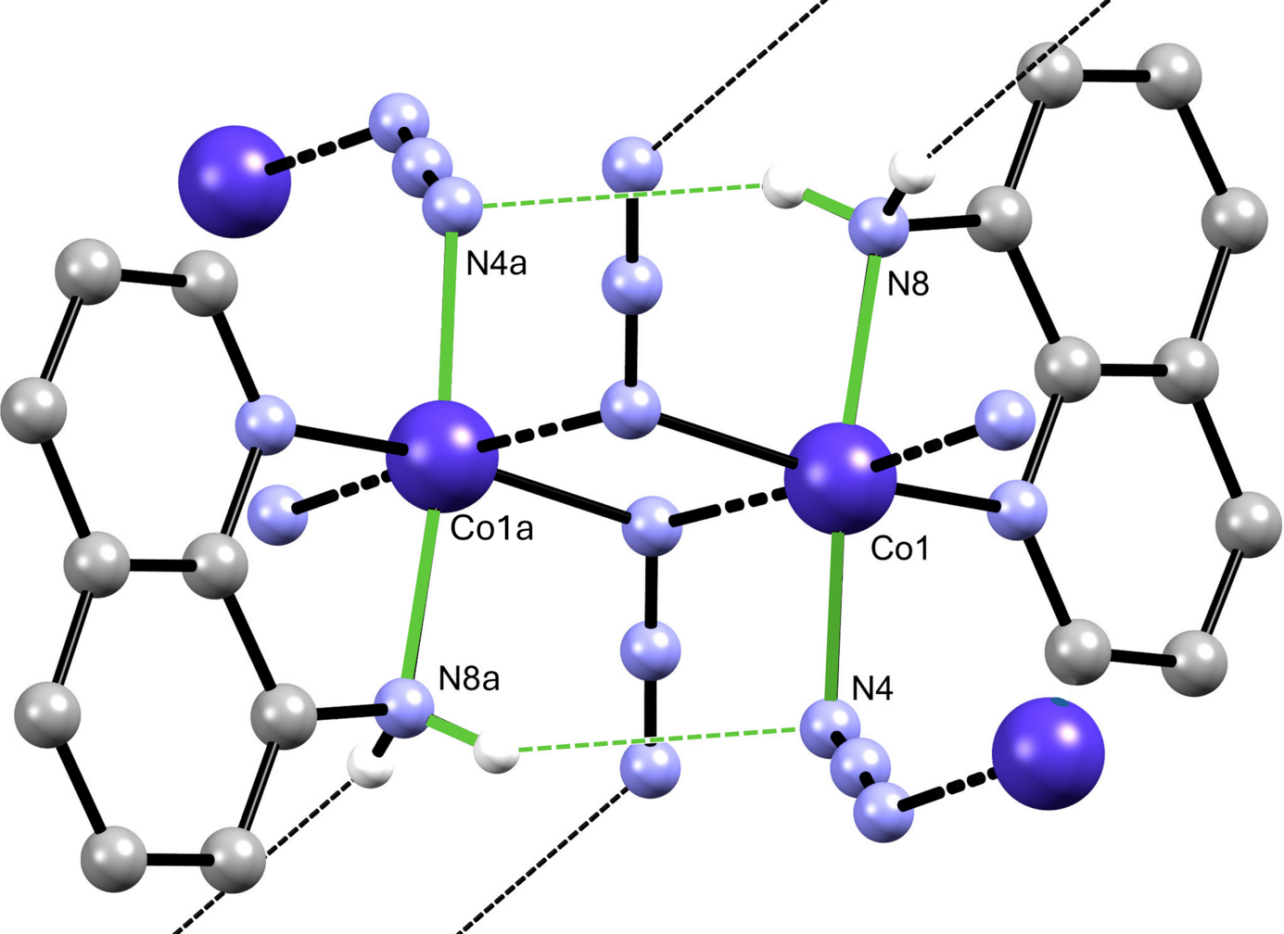
View of the intra­chain 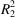
(8) N—H⋯N motif. Only H atoms involved in the hydrogen bonds are shown. Symmetry code: (*a*) −*x*, −*y* + 1, −*z* + 1.

N—H⋯N hydrogen-bonding inter­actions are observed in the extended structure (Table 2 ▸, Fig. 3 ▸). Within individual chains, 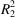
(8) hydrogen-bonded rings are observed (Fig. 4 ▸). The polymeric chains are joined by hydrogen-bonding bridges involving the 8-amino substituent on the quinoline ligands and the terminal nitro­gen atom of the μ-(1,1-azide) ligands of adjacent chains, resulting in sheets parallel to (002) containing N—H⋯N hydrogen-bond-derived 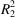
(12) inter­chain rings, as seen in Figs. 3 ▸ and 5 ▸.

## Synthesis and crystallization

The title compound was prepared solvothermally under autogenous pressure from a mixture of cobalt(II) sulfate hepta­hydrate (28 mg, 0.1 mmol), 8-amino­quinoline (14 mg, 0.1 mmol) and sodium azide (13 mg, 0.2 mmol) in a mixture of water and ethanol (3:1 *v*/*v*, 20 ml). This mixture was sealed in a Teflon-lined autoclave and held at 393 K for 2 days, and then cooled to ambient temperature at a rate of 10 K h^−1^ to give the product (yield 38%).

## Refinement

Crystal data, data collection and refinement details are summarized in Table 3 ▸.

## Supplementary Material

Crystal structure: contains datablock(s) global, I. DOI: 10.1107/S2414314624008496/bt4154sup1.cif

Structure factors: contains datablock(s) I. DOI: 10.1107/S2414314624008496/bt4154Isup2.hkl

Supporting information file. DOI: 10.1107/S2414314624008496/bt4154Isup3.mol

CCDC reference: 2380120

Additional supporting information:  crystallographic information; 3D view; checkCIF report

## Figures and Tables

**Figure 5:**
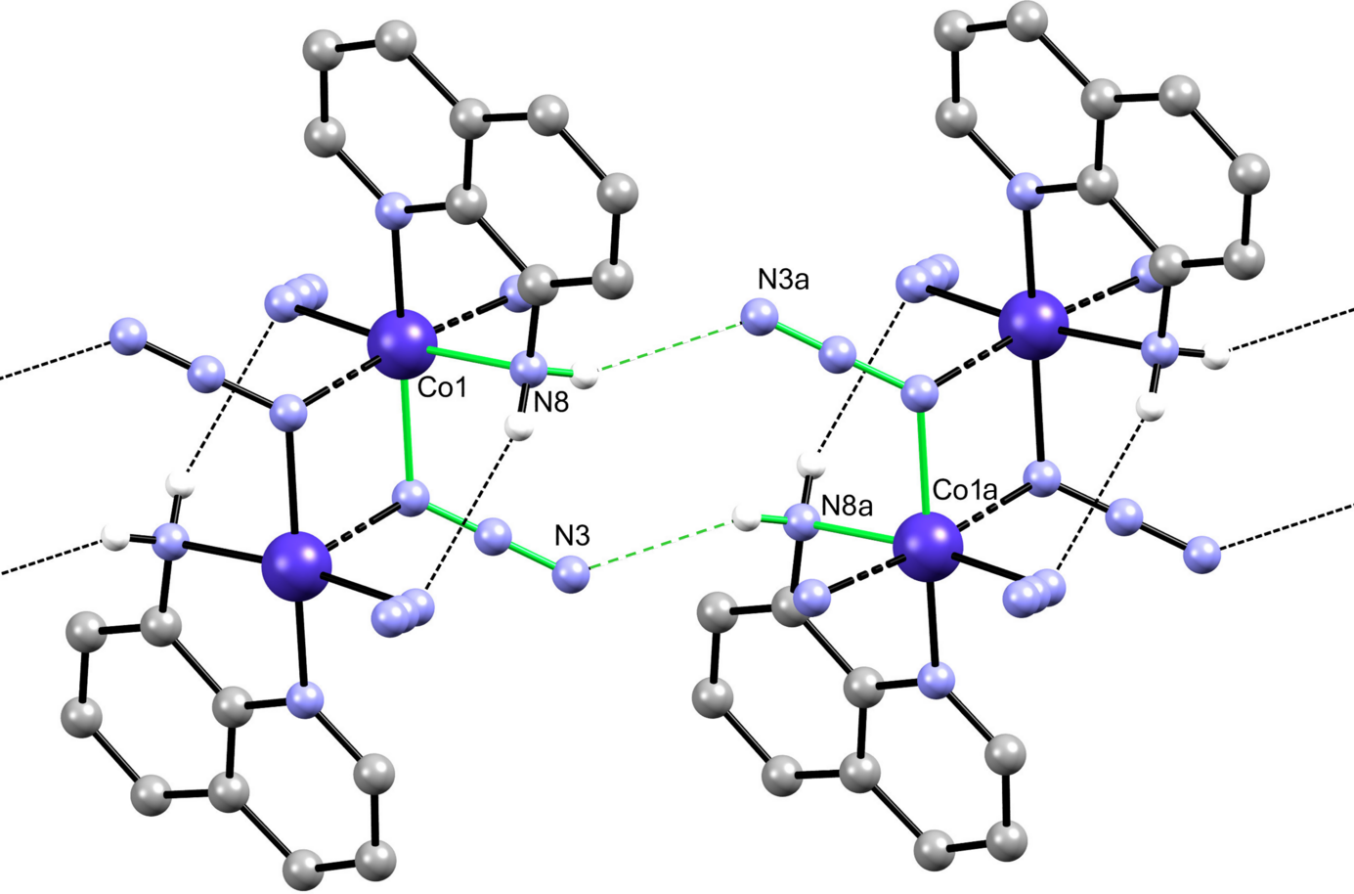
View of the inter­chain 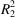
(12) N—H⋯N motif. Only H atoms involved in hydrogen bonding are shown. Symmetry code: (*a*) −*x*, −*y*, −*z* + 1.

**Table 1:** Selected bond lengths (Å)

Co1—N1	2.1020 (13)	Co1—N8	2.1684 (13)
Co1—N7	2.1100 (12)	Co1—N6^i^	2.1685 (15)
Co1—N4	2.1222 (17)	Co1—N1^ii^	2.2047 (12)

Symmetry codes: (i) 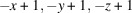; (ii) 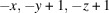.

**Table 2:** Hydrogen-bond geometry (Å, °)

*D*—H⋯*A*	*D*—H	H⋯*A*	*D*⋯*A*	*D*—H⋯*A*
N8—H8*A*⋯N3^iii^	0.84 (3)	2.46 (3)	3.218 (2)	151 (2)
N8—H8*B*⋯N4^ii^	0.88 (3)	2.73 (3)	3.502 (3)	148 (2)

Symmetry codes: (ii) 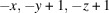; (iii) 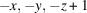.

**Table 3:** Experimental details

Crystal data	
Chemical formula	[Co(N_3_)_2_(C_9_H_8_N_2_)]
*M*_r_	287.16
Crystal system, space group	Triclinic, *P* 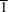
Temperature (K)	301
*a*, *b*, *c* (Å)	7.3526 (10), 8.3354 (13), 10.4053 (17)
α, β, γ (°)	97.221 (6), 102.413 (6), 111.334 (5)
*V* (Å^3^)	565.36 (15)
*Z*	2
Radiation type	Mo *K*α
μ (mm^−1^)	1.51
Crystal size (mm)	0.35 × 0.30 × 0.22

Data collection	
Diffractometer	Xcalibur CCD Sapphire3
Absorption correction	Multi-scan (*CrysAlis PRO*; Agilent, 2014 ▸)
*T*_min_, *T*_max_	0.769, 1.000
No. of measured, independent and observed [*I* > 2σ(*I*)] reflections	29909, 4356, 3882
*R*_int_	0.033
(sin θ/λ)_max_ (Å^−1^)	0.773

Refinement	
*R*[*F*^2^ > 2σ(*F*^2^)], *wR*(*F*^2^), *S*	0.031, 0.088, 1.06
No. of reflections	4356
No. of parameters	171
H-atom treatment	H atoms treated by a mixture of independent and constrained refinement
Δρ_max_, Δρ_min_ (e Å^−3^)	0.72, −0.33

Computer programs: *CrysAlis CCD* and *CrysAlis RED* (Oxford Diffraction, 2009 ▸), *SHELXS97* (Sheldrick, 2008 ▸), *SHELXL2014/7* (Sheldrick, 2015 ▸), *ShelXle* (Hübschle *et al.*, 2011 ▸) and *publCIF* (Westrip, 2010 ▸).

## full crystallographic data

### catena-Poly[[(8-aminoquinoline)cobalt(II)]-di-µ-azido-κ4N1:N3-[(8-aminoquinoline)cobalt(II)]-di-µ-azido-κ4N1:N1] Crystal data

**Table:**

[Co(N_3_)_2_(C_9_H_8_N_2_)]	*Z* = 2
*M**_r_* = 287.16	*F*(000) = 290
Triclinic, *P*1	*D*_x_ = 1.687 Mg m^−^^3^
*a* = 7.3526 (10) Å	Mo *K*α radiation, λ = 0.71073 Å
*b* = 8.3354 (13) Å	Cell parameters from 4847 reflections
*c* = 10.4053 (17) Å	θ = 4.2–32.3°
α = 97.221 (6)°	µ = 1.51 mm^−^^1^
β = 102.413 (6)°	*T* = 301 K
γ = 111.334 (5)°	Block, purple
*V* = 565.36 (15) Å^3^	0.35 × 0.30 × 0.22 mm

### catena-Poly[[(8-aminoquinoline)cobalt(II)]-di-µ-azido-κ4N1:N3-[(8-aminoquinoline)cobalt(II)]-di-µ-azido-κ4N1:N1] Data collection

**Table:**

Xcalibur CCD, Sapphire3 diffractometer	4356 independent reflections
Radiation source: fine-focus sealed tube	3882 reflections with *I* > 2σ(*I*)
Graphite monochromator	*R*_int_ = 0.033
ω scans	θ_max_ = 33.3°, θ_min_ = 2.7°
Absorption correction: multi-scan (CrysAlis PRO; Agilent, 2014)	*h* = −11→11
*T*_min_ = 0.769, *T*_max_ = 1.000	*k* = −12→12
29909 measured reflections	*l* = −16→16

### catena-Poly[[(8-aminoquinoline)cobalt(II)]-di-µ-azido-κ4N1:N3-[(8-aminoquinoline)cobalt(II)]-di-µ-azido-κ4N1:N1] Refinement

**Table:**

Refinement on *F*^2^	0 restraints
Least-squares matrix: full	Hydrogen site location: mixed
*R*[*F*^2^ > 2σ(*F*^2^)] = 0.031	H atoms treated by a mixture of independent and constrained refinement
*wR*(*F*^2^) = 0.088	*w* = 1/[σ^2^(*F*_o_^2^) + (0.0409*P*)^2^ + 0.1945*P*] where *P* = (*F*_o_^2^ + 2*F*_c_^2^)/3
*S* = 1.06	(Δ/σ)_max_ = 0.001
4356 reflections	Δρ_max_ = 0.72 e Å^−^^3^
171 parameters	Δρ_min_ = −0.33 e Å^−^^3^

### catena-Poly[[(8-aminoquinoline)cobalt(II)]-di-µ-azido-κ4N1:N3-[(8-aminoquinoline)cobalt(II)]-di-µ-azido-κ4N1:N1] Special details

**Table:**

Geometry. All esds (except the esd in the dihedral angle between two l.s. planes) are estimated using the full covariance matrix. The cell esds are taken into account individually in the estimation of esds in distances, angles and torsion angles; correlations between esds in cell parameters are only used when they are defined by crystal symmetry. An approximate (isotropic) treatment of cell esds is used for estimating esds involving l.s. planes.
Refinement. H atoms bonded to C were refined using a riding model, H atoms bonded to N were freely refined.

### catena-Poly[[(8-aminoquinoline)cobalt(II)]-di-µ-azido-κ4N1:N3-[(8-aminoquinoline)cobalt(II)]-di-µ-azido-κ4N1:N1] Fractional atomic coordinates and isotropic or equivalent isotropic displacement parameters (Å^2^)

**Table:**

	*x*	*y*	*z*	*U*_iso_*/*U*_eq_	
Co1	0.19673 (3)	0.48517 (2)	0.59913 (2)	0.03117 (6)	
N1	0.0070 (2)	0.37549 (18)	0.40029 (12)	0.0377 (3)	
N2	−0.0569 (3)	0.21922 (19)	0.35544 (13)	0.0432 (3)	
N3	−0.1176 (4)	0.0705 (2)	0.3110 (2)	0.0744 (6)	
N4	0.4085 (3)	0.7020 (2)	0.5489 (2)	0.0668 (6)	
N5	0.5181 (2)	0.68214 (18)	0.48714 (14)	0.0386 (3)	
N6	0.6244 (3)	0.6686 (2)	0.42249 (18)	0.0515 (4)	
N7	0.30637 (18)	0.58191 (15)	0.81040 (12)	0.0309 (2)	
N8	0.0067 (2)	0.26670 (17)	0.66848 (13)	0.0351 (2)	
H8A	0.023 (4)	0.175 (3)	0.642 (2)	0.057 (7)*	
H8B	−0.122 (4)	0.246 (4)	0.635 (3)	0.069 (8)*	
C1	0.2139 (2)	0.47062 (17)	0.88451 (13)	0.0291 (2)	
C2	0.0569 (2)	0.30410 (18)	0.81392 (14)	0.0313 (2)	
C3	−0.0357 (3)	0.1882 (2)	0.88582 (19)	0.0423 (3)	
H3	−0.1379	0.0784	0.8402	0.051*	
C4	0.0224 (3)	0.2336 (3)	1.0283 (2)	0.0528 (5)	
H4	−0.0415	0.153	1.0754	0.063*	
C5	0.1714 (3)	0.3946 (3)	1.09811 (17)	0.0485 (4)	
H5	0.2072	0.4232	1.192	0.058*	
C6	0.2705 (2)	0.5171 (2)	1.02765 (14)	0.0380 (3)	
C7	0.4267 (3)	0.6859 (3)	1.09254 (17)	0.0472 (4)	
H7	0.4694	0.7209	1.1863	0.057*	
C8	0.5135 (3)	0.7961 (2)	1.01716 (19)	0.0477 (4)	
H8	0.6143	0.9081	1.0587	0.057*	
C9	0.4499 (2)	0.7397 (2)	0.87556 (17)	0.0399 (3)	
H9	0.5114	0.8166	0.8252	0.048*	

### catena-Poly[[(8-aminoquinoline)cobalt(II)]-di-µ-azido-κ4N1:N3-[(8-aminoquinoline)cobalt(II)]-di-µ-azido-κ4N1:N1] Atomic displacement parameters (Å^2^)

**Table:**

	*U*^11^	*U*^22^	*U*^33^	*U*^12^	*U*^13^	*U*^23^
Co1	0.03912 (11)	0.03282 (10)	0.02878 (9)	0.01827 (8)	0.01518 (7)	0.01036 (7)
N1	0.0545 (7)	0.0401 (6)	0.0290 (5)	0.0302 (6)	0.0127 (5)	0.0079 (4)
N2	0.0667 (9)	0.0457 (7)	0.0283 (5)	0.0350 (7)	0.0140 (6)	0.0078 (5)
N3	0.1122 (18)	0.0450 (9)	0.0614 (11)	0.0375 (10)	0.0127 (11)	−0.0016 (8)
N4	0.0864 (13)	0.0440 (8)	0.0944 (14)	0.0265 (8)	0.0690 (12)	0.0229 (9)
N5	0.0422 (6)	0.0372 (6)	0.0418 (6)	0.0172 (5)	0.0174 (5)	0.0138 (5)
N6	0.0596 (9)	0.0660 (10)	0.0586 (9)	0.0427 (8)	0.0341 (8)	0.0322 (8)
N7	0.0330 (5)	0.0299 (5)	0.0327 (5)	0.0154 (4)	0.0094 (4)	0.0088 (4)
N8	0.0384 (6)	0.0306 (5)	0.0355 (6)	0.0125 (5)	0.0118 (5)	0.0065 (4)
C1	0.0347 (6)	0.0331 (6)	0.0295 (5)	0.0216 (5)	0.0125 (4)	0.0111 (4)
C2	0.0363 (6)	0.0321 (6)	0.0358 (6)	0.0195 (5)	0.0169 (5)	0.0137 (5)
C3	0.0457 (8)	0.0407 (7)	0.0561 (9)	0.0227 (6)	0.0275 (7)	0.0249 (7)
C4	0.0661 (11)	0.0702 (12)	0.0570 (10)	0.0443 (10)	0.0402 (9)	0.0426 (10)
C5	0.0646 (10)	0.0722 (12)	0.0359 (7)	0.0475 (10)	0.0247 (7)	0.0257 (8)
C6	0.0479 (8)	0.0513 (8)	0.0298 (6)	0.0352 (7)	0.0123 (5)	0.0115 (6)
C7	0.0541 (9)	0.0592 (10)	0.0331 (7)	0.0383 (8)	0.0005 (6)	−0.0009 (6)
C8	0.0439 (8)	0.0418 (8)	0.0489 (9)	0.0220 (7)	−0.0035 (7)	−0.0050 (7)
C9	0.0375 (7)	0.0325 (6)	0.0465 (8)	0.0138 (5)	0.0073 (6)	0.0065 (6)

### catena-Poly[[(8-aminoquinoline)cobalt(II)]-di-µ-azido-κ4N1:N3-[(8-aminoquinoline)cobalt(II)]-di-µ-azido-κ4N1:N1] Geometric parameters (Å, º)

**Table:**

Co1—N1	2.1020 (13)	N8—H8B	0.88 (3)
Co1—N7	2.1100 (12)	C1—C2	1.418 (2)
Co1—N4	2.1222 (17)	C1—C6	1.4186 (19)
Co1—N8	2.1684 (13)	C2—C3	1.371 (2)
Co1—N6^i^	2.1685 (15)	C3—C4	1.411 (3)
Co1—N1^ii^	2.2047 (12)	C3—H3	0.93
N1—N2	1.2012 (19)	C4—C5	1.368 (3)
N1—Co1^ii^	2.2046 (12)	C4—H4	0.93
N2—N3	1.147 (2)	C5—C6	1.410 (3)
N4—N5	1.176 (2)	C5—H5	0.93
N5—N6	1.161 (2)	C6—C7	1.417 (3)
N6—Co1^i^	2.1685 (15)	C7—C8	1.355 (3)
N7—C9	1.3273 (19)	C7—H7	0.93
N7—C1	1.3653 (18)	C8—C9	1.407 (2)
N8—C2	1.4427 (19)	C8—H8	0.93
N8—H8A	0.84 (3)	C9—H9	0.93
			
N1—Co1—N7	163.50 (5)	Co1—N8—H8B	110.0 (18)
N1—Co1—N4	94.66 (8)	H8A—N8—H8B	108 (2)
N7—Co1—N4	96.60 (7)	N7—C1—C2	117.90 (12)
N1—Co1—N8	90.80 (5)	N7—C1—C6	122.02 (13)
N7—Co1—N8	78.65 (5)	C2—C1—C6	120.08 (13)
N4—Co1—N8	173.93 (8)	C3—C2—C1	119.16 (14)
N1—Co1—N6^i^	93.17 (6)	C3—C2—N8	123.91 (14)
N7—Co1—N6^i^	98.59 (6)	C1—C2—N8	116.92 (12)
N4—Co1—N6^i^	91.25 (7)	C2—C3—C4	120.74 (17)
N8—Co1—N6^i^	85.77 (6)	C2—C3—H3	119.6
N1—Co1—N1^ii^	80.75 (5)	C4—C3—H3	119.6
N7—Co1—N1^ii^	87.21 (4)	C5—C4—C3	120.97 (16)
N4—Co1—N1^ii^	90.05 (7)	C5—C4—H4	119.5
N8—Co1—N1^ii^	93.47 (5)	C3—C4—H4	119.5
N6^i^—Co1—N1^ii^	173.87 (6)	C4—C5—C6	119.92 (15)
N2—N1—Co1	119.76 (10)	C4—C5—H5	120.0
N2—N1—Co1^ii^	122.01 (12)	C6—C5—H5	120.0
Co1—N1—Co1^ii^	99.25 (5)	C5—C6—C7	123.32 (15)
N3—N2—N1	179.1 (2)	C5—C6—C1	119.12 (16)
N5—N4—Co1	121.28 (13)	C7—C6—C1	117.56 (15)
N6—N5—N4	176.53 (18)	C8—C7—C6	119.57 (15)
N5—N6—Co1^i^	137.61 (13)	C8—C7—H7	120.2
C9—N7—C1	118.25 (13)	C6—C7—H7	120.2
C9—N7—Co1	126.30 (11)	C7—C8—C9	119.45 (16)
C1—N7—Co1	115.42 (9)	C7—C8—H8	120.3
C2—N8—Co1	111.12 (9)	C9—C8—H8	120.3
C2—N8—H8A	108.7 (17)	N7—C9—C8	123.12 (16)
Co1—N8—H8A	109.8 (17)	N7—C9—H9	118.4
C2—N8—H8B	109.3 (18)	C8—C9—H9	118.4
			
C9—N7—C1—C2	178.36 (12)	C3—C4—C5—C6	−0.6 (3)
Co1—N7—C1—C2	0.21 (14)	C4—C5—C6—C7	−179.72 (15)
C9—N7—C1—C6	−1.73 (19)	C4—C5—C6—C1	−0.1 (2)
Co1—N7—C1—C6	−179.88 (9)	N7—C1—C6—C5	−178.82 (12)
N7—C1—C2—C3	178.61 (12)	C2—C1—C6—C5	1.10 (19)
C6—C1—C2—C3	−1.30 (19)	N7—C1—C6—C7	0.79 (19)
N7—C1—C2—N8	−0.50 (17)	C2—C1—C6—C7	−179.29 (12)
C6—C1—C2—N8	179.58 (12)	C5—C6—C7—C8	−179.68 (15)
Co1—N8—C2—C3	−178.54 (11)	C1—C6—C7—C8	0.7 (2)
Co1—N8—C2—C1	0.52 (15)	C6—C7—C8—C9	−1.3 (2)
C1—C2—C3—C4	0.6 (2)	C1—N7—C9—C8	1.2 (2)
N8—C2—C3—C4	179.60 (14)	Co1—N7—C9—C8	179.11 (11)
C2—C3—C4—C5	0.4 (3)	C7—C8—C9—N7	0.3 (2)

Symmetry codes: (i) −*x*+1, −*y*+1, −*z*+1; (ii) −*x*, −*y*+1, −*z*+1.

### catena-Poly[[(8-aminoquinoline)cobalt(II)]-di-µ-azido-κ4N1:N3-[(8-aminoquinoline)cobalt(II)]-di-µ-azido-κ4N1:N1] Hydrogen-bond geometry (Å, º)

**Table:**

*D*—H···*A*	*D*—H	H···*A*	*D*···*A*	*D*—H···*A*
N8—H8*A*···N3^iii^	0.84 (3)	2.46 (3)	3.218 (2)	151 (2)
N8—H8*B*···N4^ii^	0.88 (3)	2.73 (3)	3.502 (3)	148 (2)

Symmetry codes: (ii) −*x*, −*y*+1, −*z*+1; (iii) −*x*, −*y*, −*z*+1.
